# Type 2 diabetes patients’ and providers’ differing perspectives on medication nonadherence: a qualitative meta-synthesis

**DOI:** 10.1186/s12913-015-1174-8

**Published:** 2015-11-23

**Authors:** Francesca Brundisini, Meredith Vanstone, Danielle Hulan, Deirdre DeJean, Mita Giacomini

**Affiliations:** Department of Clinical Epidemiology and Biostatistics, McMaster University, 1280 Main Street West, Hamilton, ON L8S 4 K1 Canada

**Keywords:** Systematic review, Qualitative Meta-synthesis, Medication adherence, Type 2 diabetes mellitus, Patient-centered care

## Abstract

**Background:**

Poor adherence to medication regimens increases adverse outcomes for patients with Type 2 diabetes. Improving medication adherence is a growing priority for clinicians and health care systems. We examine the differences between patient and provider understandings of barriers to medication adherence for Type 2 diabetes patients.

**Methods:**

We searched systematically for empirical qualitative studies on the topic of barriers to medication adherence among Type 2 diabetes patients published between 2002–2013; 86 empirical qualitative studies qualified for inclusion. Following qualitative meta-synthesis methods, we coded and analyzed thematically the findings from studies, integrating and comparing findings across studies to yield a synthetic interpretation and new insights from this body of research.

**Results:**

We identify 7 categories of barriers: (1) emotional experiences as positive and negative motivators to adherence, (2) intentional non-compliance, (3) patient-provider relationship and communication, (4) information and knowledge, (5) medication administration, (6) social and cultural beliefs, and (7) financial issues. Patients and providers express different understandings of what patients require to improve adherence. Health beliefs, life context and lay understandings all inform patients’ accounts. They describe barriers in terms of difficulties adapting medication regimens to their lifestyles and daily routines. In contrast, providers' understandings of patients poor medication adherence behaviors focus on patients’ presumed needs for more information about the physiological and biomedical aspect of diabetes.

**Conclusions:**

This study highlights key discrepancies between patients’ and providers’ understandings of barriers to medication adherence. These misunderstandings span the many cultural and care contexts represented by 86 qualitative studies. Counseling and interventions aimed at improving medication adherence among Type 2 diabetes might become more effective through better integration of the patient’s perspective and values concerning adherence difficulties and solutions.

## Background

Medication adherence plays an important role in the clinical care of Type 2 diabetes because it directly contributes to the effectiveness of patients’ treatment and wellbeing [1, 2]. Diabetes affects a growing number of patients, and represents one of the primary causes of death among adult individuals [3, 4]. Diabetes affects about 382 million people worldwide, of which 85 % to 95 % accountable to Type 2 diabetes in high-income countries, as well as in low-and-middle income countries [4]. The prevalence of Type 2 diabetes grows steadily, due to environmental and behavioural factors such as economic growth, urbanization, ageing populations, poor dietary habits, and decreased physical activity [4, 5].

Diabetes is a disease with no specific cure and a demanding self-management regimen [4, 5]. It is a progressive condition that requires continuous management as well as patient and provider collaboration in order to avoid both short-term and long-term life-threatening complications [4, 5]. Diabetes management targets optimal blood glucose levels, thereby preventing the onset and progression of diabetes-related complications including cardiovascular complications, nerve damage, kidney failure, eye disease, and diabetic foot, all factors that can eventually lead to death [3–5]. Effective Type 2 diabetes management can include adherence to medication regimens (hypoglycaemic oral tablets and/or insulin injections), as well as adjustment of specific life-style behaviours, such as increased physical activity, adherence to specific dietary regimens, smoking cessation, and strict monitoring of blood glucose levels [1, 5].

Although good glycemic control can help to prevent such complications, diabetes treatment regimens can be complex. Patients often do not adhere to medication regimens [1, 2, 6–12]. Non-adherence represents burdens both for patients and for healthcare systems by increasing morbidity and mortality, reducing quality of life, and raising healthcare costs [1, 2, 6, 9–11]. Traditionally, non-adherence behaviours stem from a patient’s failure or refusal to comply with the prescribed medication instructions due to a lack of knowledge or lack of motivation [7, 9–11, 13]. In this tradition, researchers investigate why patients failed to comply with providers’ recommendations [7, 13–15]. However, new perspectives on this topic acknowledge the beneficial effects on treatment outcomes of a more collaborative relationship between patient and provider that focuses on *concordance* rather than adherence or compliance with medication regimens. This perspective recognizes adherence as resulting from a broad set of factors, and linked to more than just knowledge and motivation [7, 10, 13, 16]. The shift towards a more patient-centered model of care recognizes the “empowered autonomy” of patients as equal and active partners in care, contributing experiential knowledge to the decision-making process of care [7, 10, 13, 16]. A patient-centered approach, then, encourages the use of a negotiated model of care to foster concordant treatment behaviours [7, 9–11, 13, 16].

Acknowledging patients’ voices in the treatment decision-making process requires deeper understanding of patients’ views of medications, and how these might differ from the assumptions or values of healthcare providers. This manuscript synthesizes numerous qualitative studies to distil broadly relevant and applicable insights into better medication adherence. We focus on patient and provider perceptions of patients’ barriers to medication adherence, amongst community-dwelling adults with Type 2 diabetes. In particular, our research question asks: *what barriers to medication adherence Type 2 diabetes patients and their providers identify?* This synthesis includes 73 studies which include patient perspectives, 9 studies which include provider perspectives and 4 studies which include both patient and provider perspectives. Findings reveal the full spectrum of barriers and facilitators patients face in using diabetes medications as directed. The four existing studies comparing both patient and providers perspectives highlight some key incongruencies in attitudes and perceptions towards medication adherence barriers [17–20]. Research findings reveal discrepancies between providers’ conceptualization of quality of health as opposed to the patient’s idea of overall well-being, as well as different attitudes to the risk of medication adverse effects [17–20]. However, most of these studies address particular ethnic populations, or patient populations with specific co-morbid conditions, or specific healthcare professional services, without providing an overall picture of the differences between patients and providers. This study adds to the under-researched literature on the differing perspectives on medication adherence between patients and providers. Further, analysis of the differences between patient and provider perspectives highlights areas for developing more patient-centered practices to improve medication adherence.

The topic of this study was informed by the Ontario Health Technology Advisory Committee’s Expert Advisory Panel on Community Care for Type 2 Diabetes project on the improvement of access to, and quality of, diabetes services and care to enhance prevention and improving diabetes management. This agency commissioned a report on patient perspectives on barriers and facilitators to medication adherence. During our analysis of this data, we noted the discrepancies between patient and provider perspectives and so returned to our data to perform a secondary analysis on the current topic.

## Methods

We provided the methods for the search in detail in a technical report written with the same data on medication adherence among Type 2 diabetes patients for the Ontario Health Technology Advisory Committee’s Expert Advisory Panel [21]. The report focused only on patients’ perspectives to barriers and facilitators of medication adherence. We summarize those methods here.

### Literature search

**Fig. 1:**
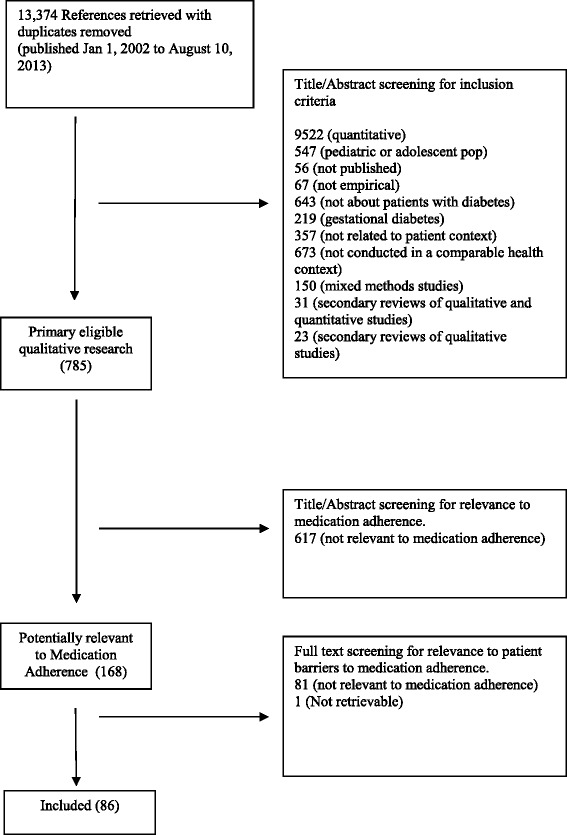
Flow diagram selection process

Figure 1 summarizes the systematic bibliographic search process. We developed a search filter that combined existing published qualitative filters [22–24] with a diabetes-topic-specific filter. Because qualitative methodology filters have poor specificity, we used exclusionary terms to improve the precision of the filter (we describe details elsewhere, available as a CHEPA Working Paper online at http://www.chepa.org/research-papers/working-papers or from corresponding author upon request) [21]. We searched OVID MEDLINE, EBSCO Cumulative Index to Nursing, Allied Health Literature (CINAHL), and ISI Web of Science Social Sciences Citation Index (SSCI), for studies published from January 1, 2002 to August 10, 2013. 2002 was chosen to produce a manageable number of results, and to reflect that the knowledge before this time was well summarized in the WHO’s 2003 report on medication adherence [1]. We included papers in English, available online through McMaster University’s library system, reporting primary qualitative empirical research, involved or addressed adults with Type 2 diabetes mellitus (including papers with both Type 1 and Type 2 diabetes), and conducted in Canada, the USA, Europe, Australia, or New Zealand. These countries were chosen because they have similar levels of resource availability (e.g. diabetes health care, medications) to Canada. When papers were not available through the library system of our large, research-intensive university we made attempts to contact the authors to request a copy of the paper through information available in the abstract/citation or a Google search. Only 1 paper was unavailable after these attempts (as shown in Fig. 1). We excluded papers that were unpublished (e.g., reports, theses), not in English, reported secondary or non-empirical studies, used non-qualitative methods, or were off-topic (that is, not addressing the topic of medication adherence). Our search terms were designed to find qualitative studies about diabetes; further refinements of the search (e.g. topic of medication adherence, like health care context) were performed manually. Examples of exclusionary terms include “coefficient” and “*p* value”. At least two reviewers independently reviewed titles, abstracts, and later full papers to determine eligibility. We reviewed titles and abstracts to identify findings related to medication adherence, medication and self-management. We then reviewed the full text of the papers before inclusion to identify any findings related to medication adherence. Data extraction was performed by two authors; all authors participated in analysis. Discrepancies were resolved through conversation between the two authors with a third author participating when an additional perspective was needed. Studies that included either Type 2 diabetes patients OR both Type 1 and Type 2 diabetes patients were included. When analyzing studies that included participants with both types of diabetes, we considered the data related to Type 2 diabetes patients when the authors provided this separately. When no distinction was made between the data from Type 1 and 2 participants, we included all data. We included a total of 86 papers in this synthesis, summarized in Table 1. For a detailed list and description of the main focus of each study see Table 2.

**Table 1:** Descriptive summary of included studies (*N* = 86)

Geography	N	Percent
Australia	5	5.8
Canada	7	8.1
Ontario	6	6.9
British Columbia	1	1.2
Europe	31	36
Netherlands	5	5.8
Romania	2	2.3
Sweden	2	2.3
United Kingdom	16	18.6
Other^a^	6	6.9
United States	43	50
Study Participants	N	%
Patients only	72	83.7
Patients and providers	5	5.8
Providers only	9	10.5
Qualitative Methodologies	N	%
Content analysis	7	8.1
Ethnography	4	4.7
Grounded theory	17	19.8
Other^b^	8	9.3
Phenomenological	4	4.7
Qualitative (otherwise unspecified)	46	53.4

^a^ “Other” countries include: Multi-country studies, Germany, Norway, Belgium, Croatia

^b^ “Other” methods include: linguistic analysis (1), discourse analysis (1), narrative analysis (1), participatory action (1), framework analysis (1), and cognitive task analysis (3)

**Table 2:** Detailed list and description of the main focus of each study

AUTHOR	DATE	TITLE	COUNTRY	METHODOLOGY	PARTICIPANTS	MAIN RESEARCH QUESTION
Ab et al.	2009	Reasons of general practitioners for not prescribing lipid-lowering medication to patients with diabetes: a qualitative study	Netherlands	Qualitative; Interviews	7 family physicians	What factors underlie GPs’ decisions not to prescribe lipid-lowering medications to patients with T2DM?
Adili et al.	2012	Inside the PAR group: The group dynamics of women learning to live with diabetes	Australia	Qualitative (participatory action research); Interviews, group discussion	11 patients with T2DM, women, older population	What is the value of group learning in helping women to live with T2DM?
Agarwal et al.	2008	GPs’ approach to insulin prescribing in older patients: a qualitative study	Ontario, Canada	Qualitative (grounded theory); Interviews	21 family physicians	What are the themes that reflect factors that influence the prescribing of insulin when treating older patients with T2DM?
Barko et al.	2011	Perceptions of diabetes symptoms and self-management strategies: a cross-cultural comparison	USA	Qualitative (descriptive); Interviews	20 patients with T2DM, Slavic immigrants and White non-immigrants, women, older population	What are the similarities and differences between perceived symptoms of T2DM and self-management strategies for Russian-speaking Slavic immigrant American women and non-Hispanic, non-immigrant White American women?
Barton et al.	2005	The diabetes experiences of Aboriginal people living in a rural Canadian community	Canada	Qualitative (descriptive); Interviews	8 patients with T2DM, Aboriginal	What are the experiences of Nuxalk people living with the challenges of T2DM, and how can these experiences inform health services in culturally specific ways?
Bhattacharya et al.	2012	Psychosocial Impacts of Type 2 Diabetes Self-Management in a Rural African-American Population	USA	Qualitative (grounded theory); Interviews	31 patients with T2DM, African American	What are participant motivations for making health behavior changes?
Bhattacharya et al.	2012b	Self-management of type 2 diabetes among African Americans in the Arkansas Delta: a strengths perspective in social-cultural context	USA	Qualitative (Grounded theory); Interviews	31 patients with T2DM, African American	What are the underlying factors influencing the promotion of T2DM?
Bissell et al.	2004	From compliance to concordance: barriers to accomplishing a re-framed model of health care interactions	UK	Qualitative (Grounded theory); Interviews	21 patients, Pakistani	What are the barriers to accomplishing a re-framed model of interactions between HPs and patients?
Bogatean et al.	2004	People with type 2 diabetes facing the reality of starting insulin therapy: factors involved in psychological insulin resistance	Romania	Qualitative (phenomenology); Interviews	18 patients with T2DM	What are the factors involved in psychological insulin resistance?
Borgsteede et al.	2011	Factors related to high and low levels of drug adherence according to patients with type 2 diabetes	Netherlands	Qualitative; Interviews	20 patients with T2DM	What are the factors related to high and low levels of drug adherence according to patients with T2DM in primary care?
Borovoy Hine	2008	Managing the unmanageable: elderly Russian Jewish émigrés and the biomedical culture of diabetes care	USA	Qualitative; Interviews	13 patients with T2DM, elderly Russian Jewish émigrés; 2 healthcare providers; 5 other	What is the apparent resistance of elderly Russian Jewish émigrés to the dominant U.S. biomedical model of diabetes treatment?
Broom & Whittaker	2004	Controlling diabetes, controlling diabetics: moral language in the management of diabetes type 2	Australia	Qualitative; Interviews	119 patients with T2DM; 56 service providers	How is moral identity negotiated (through a language of control, surveillance, discipline, and responsibility) in the efforts to integrate, live with, and control T2DM?
Brown, J et al.	2002	The role of patient, physician and systemic factors in the management of type 2 diabetes mellitus	Ontario, Canada	Qualitative; Focus groups	30 Family physicians	What are the contextual dimensions and subsequent interactions that contribute to a lack of adherence in the application of guidelines for T2DM?
Brown, K et al.	2007	Health beliefs of African-Caribbean people with type 2 diabetes: a qualitative study	UK	Qualitative; Interviews	16 patients with T2DM, African-Caribbean	How do health beliefs influence the way African–Caribbean people with T2DM manage their illness?
Burke et al.	2006	Patients with diabetes speak: Exploring the implications ofpatients’ perspectives for their diabetes appointments	USA	Qualitative (grounded theory); Focus groups	8 patients with T2DM	How might physicians use information about patients’ perspectives to improve patients’ self-management of T2DM and thereby their glycemic control?
Cardol et al.	2012	People with mild to moderate intellectual disability talking about their diabetes and how they manage	Netherlands	Qualitative; Interviews	17 patients with T1DM + T2DM, Intellectual Disability	How do people with Intellectual Disability experience having diabetes and how do they manage the condition? How can understanding this information support in the engagement of self-management activities?
Connor et al.	2012	Listening to patients’ voices: linguistic indicators related to diabetes self-management	USA	Qualitative (linguistic analysis); Interviews	43 patients with T2DM	What are the most prominent linguistic indicators of two constructs that have been found to be important factors in models of health self-management: control orientation and agency?
Coronado et al.	2004	Attitudes and beliefs among Mexican Americans about type 2 diabetes	USA	Qualitative; Focus groups	42 patients with T2DM, Mexican Americans	Knowing that Hispanics in the United States are at a disproportionately high-risk for T2DM, what are the attitudes and beliefs about diabetes among this group?
Corser et al.	2010	Contemporary Adult Diabetes Mellitus Management Perceptions	USA	Qualitative; Group interviews	44 patients with T2DM	How do patients’ self-management beliefs and practices affect the nature of key diabetes care office visit decisions?
Courtenay et al.	2010	The views of patients with diabetes about nurse prescribing.	UK	Qualitative; Interviews	41 patients with T1DM + T2DM	What are the views of patients receiving prescriptions from Nurse Practitioners and what are the advantages and disadvantages of NP’s prescribing this medication?
Feil et al.	2011	Impact of dementia on caring for patients’ diabetes	USA	Qualitative (grounded theory); Focus groups	21 caregivers of patients with co-morbid T2DM and dementia	What are caregivers’ challenges and quality-of-life issues managing diabetes in patients with dementia.
Felea et al.	2013	Perceptions of Life Burdens and of the Positive Side of Life in a Group of Elderly Patients with Diabetes: A Qualitative Analysis through Grounded Theory	Romania	Qualitative (grounded theory); Interviews	57 patients with T2DM, older population	What are the main concerns of frail elderly people diagnosed with diabetes in terms of the perception of their burdens and their distinctive views on the positive side of life?
Frandsen & Kristensen	2002	Diet and lifestyle in type 2 diabetes: the patient’s perspective	Multiple Countries	Qualitative; Group interviews	123 patients with T2DM	According to patients across four European countries and the United States, what are the issues and barriers related to diet, lifestyle, and medication adherence?
Garrett & Martin	2003	The Asheville Project: participants’ perceptions of factors contributing to the success of a patient self-management diabetes program	USA	Qualitative; Focus groups and interviews	21 patients with T1DM + T2DM; 4 pharmacists; 1 diabetes educator; 6 project managers	What are patients’, providers’, and managers’ perceptions of the factors that contributed to the success of the Asheville Project: a Patient Self-Management Diabetes Program?
Gazmararian et al.	2009	Perception of Barriers to Self-care Management Among Diabetic Patients	USA	Qualitative; Focus groups	35 patients with unspecified DM, African-American, economically disadvantaged	What are the individual, educational, and system barriers that limit low-income diabetes patients’ ability to achieve optimal diabetes self-management?
George & Thomas	2010	Lived experience of diabetes among older, rural people	USA	Qualitative (phenomenology); Interviews	10 patients with unspecified DM, elderly population, rural	What are the experiences and perceptions of self-management of diabetes as narrated by older people diagnosed with insulin-dependent diabetes living in a rural area?
Goering & Mathias	2010	Coping with chronic illness: information use and treatment adherence among people with diabetes	USA	Qualitative (content analysis); Interviews	21 patients with T2DM	How can we understand the complex relationship among information usage, medication adherence, and disease management in people with T2DM?
Gorawara-Bhat et al.	2008	Communicating with older diabetes patients: Self-management and social comparison	USA	Qualitative (grounded theory); Interviews	28 patients T2DM, elderly population	As healthcare goals and self-management behaviors are frequently shaped through social comparisons with peers/family members, what is the role of social comparison in older patients with T2DM?
Grant et al.	2011	Diabetes oral medication initiation and intensification: patient views compared with current treatment guidelines	USA	Qualitative (content analysis); Focus groups	50 patients with T2DM	What are patient perceptions about medication management principles underlying American Diabetes Association (ADA) published treatment algorithms?
Guell	2012	Self-care at the margins: meals and meters in migrants’ diabetes tactics	Germany	Ethnographic fieldwork; semi-structured interviews and participant observation	17 healthcare providers; 7 patients with T2DM, Turkish migrants	What are Turkish migrants’ everyday practices of diabetes self-management in Berlin, Germany?
Hayes et al.	2006	Understanding diabetes medications from the perspective of patients with type 2 diabetes: prerequisite to medication concordance	USA	Qualitative (content analysis); Focus groups	138 patients with T2DM	What are patient’s perceptions of T2DM treatment, specifically related to medication experiences?
Heisler et al.	2009	Participants’ Assessments of the Effects of a Community Health Worker Intervention on Their Diabetes Self-Management and Interactions with Healthcare Providers	USA	Qualitative; Interviews	40 patients with T2DM, African-American and Latino	How does the program influence participants’ diabetes care and interactions with healthcare providers, and what gaps, if any, does it address?
Helsel et al.	2005	Chronic illness and Hmong shamans	USA	Qualitative (grounded theory); Interviews	11 patients with T2DM or hypertension, Hmong Shaman	How do Hmong Shamans’ understand and manage their chronic illness, and how can this perspective be used as a gateway to understanding how the broader Hmong American community perceive these conditions?
Henderson	2010	Divergent models of diabetes among American Indian elders	USA	Qualitative (non-random intensity sample); Interviews	30 patients with T2DM, American Indian elders	What are the belief systems about diabetes in American Indian elders, and what are the effects of culture on care-seeking, adherence, and diabetes self-care?
Hinder & Greenhalgh	2012	“This does my head in”. Ethnographic study of self-management by people with diabetes	UK	Ethnographic study; Shadowing, interviews, observation	30 people with T1DM + T2DM	Why is self-management of diabetes challenging for some, and how can research produce a richer understanding of how people live with diabetes?
Ho & James	2006	Cultural barriers to initiating insulin therapy in Chinese people with type 2 diabetes living in Canada	Ontario, Canada	Qualitative (framework analysis); Interviews	5 patients with T2DM, Chinese-Canadian, insulin dependent	What are some of the cultural barriers (as influenced by factors specific to the Chinese culture) to initiating insulin therapy among Chinese individuals with T2DM living in Canada?
Holmstrom & Rosenqvist	2005	Misunderstandings about illness and treatment among patients with type 2 diabetes	Sweden	Phenomenology; Video recordings and transcribed patient reflections	18 patients with T2DM, Swedish	What are the specific misunderstandings that Swedish patients with T2DM have about their illness and treatment, and how can health care services support rather than obstruct self-care and learning?
Hornsten et al.	2011	A model of integration of illness and self-management in type 2 diabetes	Sweden	Qualitative (content analysis); Narrative interview	44 patients with T2DM, Swedish-speaking	What is the process of illness integration and self-management among people with T2DM?
Hu et al.	2012	The Meaning of Insulin to Hispanic Immigrants With Type 2 Diabetes and Their Families	USA	Qualitative (content analysis); Focus groups	43 patients with T2DM, Hispanic	What is the meaning of Insulin among a sample of Hispanic immigrants with T2DM and their family members/significant others, and what strategies and further research are necessary to dispel negative perceptions and facilitate positive experiences?
Huang et al.	2005	Self-reported goals of older patients with type 2 diabetes mellitus	USA	Qualitative (grounded theory); Interviews	28 patients with T2DM, elderly population	What are the self-reported healthcare goals, factors influencing these goals, and self-care practices of older patients with T2DM, and how can this knowledge support providers in communicating with older patients about complex medical decisions?
Hunt et al.	2012	The changing face of chronic illness management in primary care: a qualitative study of underlying influences and unintended outcomes	USA	Qualitative; Interviews and observations	58 clinicians and 70 patients with T2DM and hypertension, observations of 107 clinical consultations with 12 clinicians	Due to the recent and dramatic increase in the diagnosis and pharmaceutical management of common chronic illnesses, how can qualitative data collected in primary care clinics help assess how these trends play out in clinical care?
Jeavons et al.	2006	Patients with poorly controlled diabetes in primary care: healthcare clinicians’ beliefs and attitudes	UK	Qualitative; Focus groups	23 healthcare providers (family physicians and nurses)	What are doctors’ and nurses’ attitudes and beliefs about treating patients with T2DM with less than ideal glycemic control while receiving maximal oral treatment in primary care?
Jenkins et al.	2011	Participants’ experiences of intensifying insulin therapy during the Treating to Target in Type 2 Diabetes (4-T) trial: qualitative interview study	UK	Qualitative (grounded theory); Interviews	41 patients with T2DM, insulin dependent	What are participants’ experiences of intensifying insulin therapy during the Treating to Target in Type 2 Diabetes (4-T) trial, and specifically, how do participants’ manage anxiety around increased likelihood of injecting insulin in public places?
Klein & Lippa	2012	Assuming control after system failure: type II diabetes self-management	USA	Qualitative (cognitive task analysis); Interviews, document review, non-participant observation	Web users and interviewees with T2DM, unknown number	How do patients bridge the gap between existing education programs and the real, dynamic challenges of diabetes self-management?
Klein & Meininger	2004	Self Management of Medication and Diabetes: Cognitive Control	USA	Qualitative (cognitive task analysis); Interviews	T2DM patients, unknown number	What self-management problems do Type 2 diabetic patients face?
Lamberts et al.	2010	The role of the community pharmacist in fulfilling information needs of patients starting oral anti-diabetics	Netherlands	Qualitative; Interviews and focus groups	42 patients with T2DM	What are the information needs of patients who have recently started treatment with oral anti-diabetics and what are the opportunities for pharmacy regarding the provision of information for patients with T2DM?
Lawton et al.	2005	Perceptions and experiences of taking oral hypoglycaemic agents among people of Pakistani and Indian origin: qualitative study	UK	Qualitative (grounded theory); Interviews	32 patients with T2DM, British Indian or Pakistani	What are British Pakistani and British Indian patients’ perceptions and experiences of taking oral hypoglycemic agents (OHAs), and how does ambivalence toward Western drugs influence medication adherence?
Lawton et al.	2008	Patients’ perceptions and experiences of taking oral glucose-lowering agents: a longitudinal qualitative study	UK	Qualitative (longitudinal); Interviews	20 patients with T2DM	What are patient expectations, perceptions and experiences of oral glucose-lowering agents (OGLAs), including their reasons for taking/not taking these drugs as prescribed and what recommendations exist for developing interventions to improve OGLA adherence?
Lee et al.	2007	The development and evaluation of written medicines information for type 2 diabetes	Australia	Qualitative; Interviews	24 patients with T2DM	Using the ‘Consumer Involvement Cycle’ to investigate consumer perspectives and the need for medication information for patients with T2DM, how can this information be used to develop appropriate WMI for the T2DM population?
Lippa & Klein	2008	Portraits of patient cognition: how patients understand diabetes self-care	USA	Qualitative; Interviews	18 patients with T2DM	How do T2DM patients with low, moderate, and good glycemic control conceptualize self-care?
Lippa et al.	2008	Everyday expertise: cognitive demands in diabetes self-management	USA	Qualitative (cognitive task analysis); Interviews	18 patients with T2DM	What is the relationship between decision-making and successful diabetes self-management?
Lutfey	2005	On practices of ‘good doctoring’: reconsidering the relationship between provider roles and patient adherence	USA	Ethnography; observations of patient-practitioner consultations, Qualitative; semi-structured interviews	170 patients with unspecified DM; 25 practitioners	How do medical practitioners conceptualise, tailor their actions, and strategically enact practices with specific patients in order to maximise their adherence to treatment regimens?
Lynch et al.	2012	Concepts of diabetes self-management in Mexican American and African American low-income patients with diabetes	USA	Qualitative (grounded theory); Focus groups	84 patients with T2DM, African American and Mexican American	How do low-income minority conceptualize diabetes self-management and to what extent do patient beliefs correspond to evidence-based recommendations?
Mathew et al.	2012	Self-management experiences among men and women with type 2 diabetes mellitus: a qualitative analysis	Ontario, Canada	Qualitative; Telephone interviews and focus groups	35 patients with T2DM	What are the differences in diabetes self-management - specifically needs, barriers and challenges among men and women living with T2DM?
Mayberry & Osborn	2012	Family support, medication adherence, and glycemic control among adults with type 2 diabetes	USA	Mixed method: Qualitative; Focus groups; quantitative; Surveys	45 patients with T2DM (*n* = 61 for surveys)	Does the perception of family members’ knowledge about diabetes have a positive or negative association with patients’ diabetes-specific supportive behaviors and medical adherence?
McSharry et al.	2013	‘The chicken and egg thing’: cognitive representations and self-management of multimorbidity in people with diabetes and depression	UK	Qualitative; Interviews	17 patients with T1DM + T2DM and depression	How do patients perceive and report the impact and management of multimorbid representations of diabetes and depression?
Mishra et al.	2011	Adherence to Medication Regimens among Low-Income Patients with Multiple Comorbid Chronic Conditions	USA	Qualitative; Focus groups	50 patients with T1DM + T2DM, 40+ years of age, 2+ chronic conditions	What are the facilitators and barriers for adherence to multiple medications among low-income patients with comorbid chronic physical and mental health conditions?
Mohan et al.	2013	Illustrated medication instructions as a strategy to improve medication management among Latinos: a qualitative analysis	USA	Qualitative; Focus groups and interviews	38 patients with T2DM, Latino	What are the barriers to effective medication management for Latino patients with diabetes, and what strategies could help improve medication management among this vulnerable population?
Morris et al.	2005	Experiences of people with type 2 diabetes who have changed from oral medication to self-administered insulin injections: a qualitative study	UK	Qualitative; Interviews	6 patients with T2DM, older population	What are the lived subjective experiences, expectations, and impact for patients who have recently started insulin?
Morrow et al.	2008	Integrating diabetes self-management with the health goals of older adults: a qualitative exploration.	USA	Qualitative; Interviews	24 patients with T2DM, hypertension, and at least one other chronic comorbidity, elderly population; 10 caregivers	What are the life and health goals of older adults with diabetes, and what are the factors that influence their diabetes self-management?
Moser et al.	2008	Self-management of type 2 diabetes mellitus: a qualitative investigation from the perspective of participants in a nurse-led, shared-care programme in the Netherlands	Netherlands	Qualitative (grounded theory); Interviews	15 patients with T2DM, elderly population	How do patients with T2DM experience self-management in a nurse-led shared care program?
Nagelkerk et al.	2006	Perceived barriers and effective strategies to diabetes self-management	USA	Qualitative (content analysis); Focus groups	24 patients with T2DM, rural	What do patients perceive as barriers and effective strategies for self-management in a rural setting?
Nair et al.	2007	“I take what I think works for me”: a qualitative study to explore patient perception of diabetes treatment benefits and risks.	Ontario, Canada	Qualitative (grounded theory); Interviews	18 patients with T2DM	What is the experience of benefit and risk assessment for people with T2DM when making treatment decisions?
Noakes	2010	Perceptions of black African and African-Caribbean people regarding insulin	UK	Qualitative; Focus groups	13 patients with T2DM, African and African-Caribbean	What are black African and African-Caribbean adults’ perceptions of insulin treatment?
Onwudiwe et al.	2011	Barriers to self-management of diabetes: a qualitative study among low-income minority diabetics	USA	Qualitative; Focus groups	31 patients with T2DM, predominantly African-American, low income	What do low income minority patients perceive as barriers to self-management?
Parry et al.	2006	Issues of cause and control in patient accounts of Type 2 diabetes.	UK	Qualitative (discourse analysis); Interviews	40 patients with T2DM	How do patients view diabetes services and disease causation and management, and what are the implications of these beliefs for clinical management?
Patel et al.	2012	Insulin initiation and management in people with Type 2 diabetes in an ethnically diverse population: the healthcare provider perspective.	UK	Qualitative; Interviews	14 healthcare professionals who care for patients with T2DM	What are barriers to prescribing insulin to multi-ethnic adults (mostly South Asian setting) with T2DM?
Phillips	2007	Starting patients on insulin therapy: diabetes nurse specialist views	UK	Qualitative (exploratory); Interviews	4 diabetes nurse specialists	What are the challenges of converting patients with T2DM to insulin therapy?
Rahim-Williams	2011	Beliefs, behaviors, and modifications of type 2 diabetes self-management among African American women	USA	Qualitative; Interviews, participant observation, self-management questionnaire	25 patients with T2DM, women, African American	What are the health beliefs and behaviours affecting self-management of African American women with T2DM?
Raphael et al.	2012	A toxic combination of poor social policies and programmes, unfair economic arrangements and bad politics: the experiences of poor Canadians with Type 2 diabetes	Ontario, Canada	Qualitative; Interviews	60 patients with T2DM, low income	What are the day to day experiences of low income adults with T2DM living in poverty?
Rayman & Ellison	2004	Home alone: the experience of women with type 2 diabetes who are new to intensive control	USA	Qualitative (Grounded theory); Interviews	14 patients with T2DM, women	What are the early experiences of women learning intensive self-management of T2DM?
Renfrew et al.	2013	Barriers to Care for Cambodian Patients with Diabetes: Results from a Qualitative Study	USA	Qualitative; Focus groups	15 patients with T2DM, Cambodian; 25 clinicians; 5 bilingual Khmer staff	What are potential barriers to care for Cambodian patients with T2DM?
Rise et al.	2013	Making and Maintaining Lifestyle Changes after Participating in Group Based Type 2 Diabetes Self-Management Educations: A Qualitative Study.	Norway	Qualitative (Phenomenological); Focus groups and interviews	23 patients with T2DM	How do patients make and maintain lifestyle changes after participating in group-based self-management education for T2DM?
Shaw et al.	2013	Resources, roadblocks and turning points: a qualitative study of American Indian/Alaska Native adults with type 2 diabetes	USA	Qualitative; Focus groups and interviews	13 patients with T2DM, Alaska Native and American Indian	What are the perceived psychosocial needs and barriers to self-management for Alaskan Native and American Indian adults with T2DM?
Stack et al.	2008	A qualitative exploration of multiple medicines beliefs in co-morbid diabetes and cardiovascular disease	UK	Qualitative (modified grounded theory); Interviews	19 patients with comorbid T2DM and cardiovascular disease	What are the perceptions of multiple medications expressed by patients managing co-morbid T2DM and cardiovascular disease?
Thorlby et al.	2011	Clinicians’ views of an intervention to reduce racial disparities in diabetes outcomes	USA	Qualitative; Interviews	12 physicians; 4 nurse practitioners; 1 physician assistant	What do primary care practitioners understand about racial disparities among patients with T2DM and what are the perceptions of a cultural competency intervention?
Tjia et al.	2008	Beneath the surface: discovering the unvoiced concerns of older adults with type 2 diabetes mellitus	USA	Qualitative; Interviews	22 patients with T2DM, older population, at least 5 medications	What are the concerns of older patients with T2DM about their medication regimens?
Venkatesh & Weatherspoon	2013	Social and health care provider support in diabetes self-management.	USA	Qualitative; Interviews	30 patients with T2DM, Asian Indian immigrants	What social and health care support do Asian Indian adults with T2DM have for self-management?
Vermeire et al.	2007	Obstacles to adherence in living with type-2 diabetes: an international qualitative study using meta-ethnography (EUROBSTACLE)	Multiple country	Qualitative (meta-ethnography); Focus groups	246 patients with T2DM	What barriers do patients with T2DM encounter when adhering to treatment regimens?
Vinter-Repalust et al.	2004	Obstacles which patients with type 2 diabetes meet while adhering to the therapeutic regimen in everyday life: qualitative study	Croatia	Qualitative (content analysis); Focus groups	49 patients with T2DM	What is the experience of T2DM, what are expectations of the health care system, and what barriers to adhering to the therapeutic regimen are encountered?
Wan et al.	2012	Conceptualizations of patient empowerment among individuals seeking treatment for diabetes mellitus in an urban, public-sector clinic.	USA	Qualitative; Interviews	29 patients with T2DM	How do patients perceive patient empowerment as it applies to treatment, interactions with HPs and self-management?
Wang et al.	2012	Focus group study assessing self-management skills of Chinese Americans with type 2 diabetes mellitus	USA	Qualitative; Focus groups	24 patients with T2DM, Chinese-American	What beliefs, experiences, knowledge and skills facilitate self-management among Chinese-American adults with T2DM?
Wens et al.	2005	GPs’ perspectives of type 2 diabetes patients’ adherence to treatment: A qualitative analysis of barriers and solutions	Belgium	Qualitative (descriptive, content analysis); focus groups	40 family physicians	What are the thoughts and feelings of FPs about T2DM patient compliance/adherence?
Williams et al.	2008	Adherence to multiple, prescribed medications in diabetic kidney disease: A qualitative study of consumers’ and health professionals’ perspectives	Australia	Qualitative (descriptive exploratory); Interviews and focus groups	23 patients with T2DM and chronic kidney disease; 16 healthcare professionals	What factors affect adherence to multiple prescription medications for patients with co-morbid T2DM and diabetic kidney disease?
Wilson et al.	2013	Patient and carer experience of obtaining regular prescribed medication for chronic disease in the English National Health Service: a qualitative study	UK	Qualitative; Interviews	21 patients with T1DM + T2DM and other chronic conditions; 9 caregivers	What are patient and caregiver experiences of community and primary care services for chronic disease, especially service delivery of repeat prescriptions?
Wong et al.	2005	Perspectives on clinic attendance, medication and foot-care among people with diabetes in the Torres Strait Islands and Northern Peninsula Area	Australia	Qualitative (descriptive); Interviews and focus groups	67 patients with T2DM, Indigenous Torres Strait Islanders	What are the perspectives and needs of indigenous people with T2DM? How might successful self-management be promoted in this group?

*DM* = Diabetes Mellitus, *T1DM* = Type 1 Diabetes Mellitus, *T2DM* = Type 2 Diabetes Mellitus, *GP* = General Practitioner, *FP* = Family physician

We used the integrative technique of qualitative meta-synthesis to analyze our data [25–27]. Qualitative meta-synthesis aims to both summarize a range of findings across studies while retaining the original meaning and to compare and contrast findings across studies to produce a new integrative interpretation [28]. Analytical integrative meta-synthesis combines and synthesizes findings in new interpretative ways, while preserving the differences and complexities of the topic under study. Congruent with this meta-synthesis methodology, we started with a pre-defined topic and research question, which guided data collection, extraction of findings, and analysis. We retrieved all qualitative research relevant to this research question. Critical appraisal remains controversial for qualitative research methodology, in part because there is a lack of consensus in the field about what constitutes high quality research [29]. Procedural detail is typically under-reported, but even when reported and achieved, methodological procedures do not always guarantee to useful results [27, 29]. Accordingly, we followed current conventions in qualitative meta-synthesis and neither appraised nor excluded papers on the basis of any superficial indicators of quality other than excluding papers which did not provide evidence to support their stated findings [25, 26, 28, 30–34].

The data extraction phase involved identifying findings relevant to the topic, focusing on the authors’ secondary interpretations – i.e., the authors’ “data-driven and integrated discoveries, judgments, and/or pronouncements researchers offer about the phenomena, events, or cases under investigation” [26]. Primary data makes ad hoc appearances in qualitative reports; while we did not focus our analysis on these excerpts per se, we did extract participant quotes when useful for illustrative purposes.

We analyzed our data using a staged coding process similar to grounded theory, [35, 36] breaking findings into their component concepts and then grouping and re-grouping those findings across studies according to inductively identified themes. First, FM, MV, and DH coded the same sources of data (the 86 articles) and identified the preliminary categories. Categories were formed based on both prevalence of information across a large number of studies and usefulness or importance of information in a smaller number of studies. These categories provided the foundation for our interpretive insights of medication adherence across the body of research. We used a constant comparative and iterative approach, in which we compared preliminary categories with the research findings, raw data excerpts, and co-investigators’ interpretations of the studies. FM, MV, and DH met regularly to discuss the analytical findings and the next analytical steps. Finally, all the authors jointly negotiated the final emerging analytical themes.

All authors participated in the overall analytical process, meeting regularly to discuss the iterative process of analysis, compare findings and interpretations, and decide how to move forward.

## Results

The 86 included studies involved 2797 individuals with Type 2 diabetes, 40 caregivers, and 356 clinicians. The integrative analysis of these studies provides rich findings concerning how patients and providers perceive barriers to medication adherence. We organize these findings into 7 categories of barriers and facilitators: (1) emotional experiences as positive and negative motivators to adherence, (2) intentional non-compliance, (3) patient-provider relationship and communication, (4) information and knowledge, (5) medication administration, (6) social and cultural beliefs, and (7) financial issues. For each, we describe how patients and providers understand the barriers, and highlight key areas of congruent vs. divergent understandings.

### Emotions increasing and decreasing adherence

Both positive and negative emotions can impair or promote medication adherence. Positive emotions, such as experiencing positive health benefits of insulin treatment [37–41], can reinforce self-reported feelings of empowerment [37–41], and the ability to follow-through with self-care [40, 42–44]. Emotional and social support promote a sense of self-efficacy and commitment to lifestyle changes [22, 45–52], encouraging patients to do better and stay “on track” [46, 48–58].

Negative emotions such as fear, self-blame, guilt, shock, helplessness, and frustration can also either raise or lower adherence. Patients frightened by symptoms returning, early death, and potential complications of diabetes sometimes become more serious about medication adherence [41, 47, 58–66]. Observing the suffering of others with diabetes complications can motivate patients to adhere strictly to their own treatments [51, 59, 67].

However, some patients prefer providers to emphasize the potential benefits of adhering, rather than the risks of non-compliance [41, 43, 44, 58]. Those with increasing complications and intensifying treatment sometimes feel they have already failed at managing the disease, creating a “vicious circle of low motivation” [41, 61, 68–74]. Distress – whether from diabetes or other sources – can also demotivate medication adherence [71, 75, 76]. Co-morbid conditions, such as heart disease, hypertension, depression, kidney failures, decreasing sight [22, 44, 50, 57, 66, 68, 77–79] can also lead to stress and complicate self-management practices [17, 22, 50, 57, 58, 66, 68, 77–80]. However, co-morbidities can have the opposite effect of increasing motivation as successful self-management promotes self-confidence [44, 45, 52, 58, 81].

Healthcare professionals peripherally address the theme of emotions in conversations of motivation, explicitly attributing poor adherence to patients’ lack of motivation, even when providers do not explicitly discuss the impact of emotions on motivation [75, 82–85].

While patients’ motives are deep rooted and difficult to modify [85], providers echo patients’ perspectives on the influence of symptoms in adherence behaviours [78, 82]. Asymptomatic patients adhere less consistently to medication [58, 83, 86]. In contrast, patients who feel unwell, thus frightened, convert to insulin therapy more willingly [86]. Some providers use insulin as a threat to motivate their patients to improve adherence [41, 58]. Providers, as patients, also recognize that symptom improvement motivates patients [82], acknowledging the motivating effect of positive emotions related to empowerment and success [78].

### Intentional non-adherence

Some patients intentionally and purposefully do not follow their medication regimens. We conceptualize intentional non-adherence as the patients’ refusal to adhere to a specific medication regimen. Patients’ beliefs and attitudes toward the health care system can promote informed and not informed intentional non-compliant behaviours [17, 43, 50, 78, 87–89].

Intentional non-adherence sometimes results from denial about the seriousness of diabetes [47, 57, 65, 68, 77, 90]. Denial of the severity of diabetes may relate to the belief that “everybody’s got it” [90], or to the underlying scepticism and lack of trust about the effectiveness of the treatment coupled with the fear that the prescribed medication is unnecessary, unhealthy, or dangerous [44, 50, 80, 89, 91]. Most commonly patients decide not to adhere to medication regimens as an effort to avoid side effects [50, 67, 68, 78, 79, 89, 92–94]. This type of intentional non-compliance often takes a trial and error approach, with the patient self-adjusting medication (i.e. doses and timings) [17].

Providers in several studies describe a scenario where patients agree to take the medication, but then do not follow through for unclear reasons despite provider’s “best detective work” [75, 95]. Providers ascribe different motivations to this behaviour, including cultural motives (e.g. preference for traditional medication), financial constraints, depression, and poor cognitive ability [17, 85]. However, we found no evidence that providers recognize that intentional non-adherence may result from a patient's attempt to mitigate unpleasant medication side effects.

### Patient-provider relationship and communication

Many studies address the nature of the relationship between patients and health care providers and how this relationship affects medication adherence and self-management practices either positively or negatively [17–20, 37–44, 48–60, 62, 64–70, 72, 73, 75, 76, 78, 81, 89, 91, 93, 94, 96–109]. Patients describe their relationship with their provider in relation to several types of facilitators, including health care professionals’ support, collaboration and improved communication strategies [17–19, 38, 39, 41–44, 48–54, 57–59, 62, 64–70, 72, 73, 76, 78, 81, 93, 94, 96–105]. However, many patients remark on the disconnect between treatment recommendations and their everyday life, as well as perceptions of lack of support, communication barriers, challenges of working with culturally insensitive providers, and barriers to accessing health care providers, such as time constraints during visits [17, 18, 20, 37–40, 42–44, 48, 50, 52–56, 59, 60, 62, 64, 68–70, 72, 73, 75, 76, 78, 81, 89, 91, 93, 94, 96–99, 101, 102, 105–109]. In particular, patients describe a desire to be “perceived as persons, not illnesses” [66, 75, 81]. Without the understanding of patient’s life contextual factors, providers may set unrealistic targets, which patients deem impossible, thus frustrating [17, 44, 69, 75, 91, 99]. Patients attribute providers’ unrealistic expectations to a lack of support or disinterest, which results in feelings of distrust [17, 18, 44, 52, 59, 69, 75, 91, 96, 97, 99, 107]. Many patients report hope for a collaborative relationship based on mutual trust and agreement between patient and provider, which would allow them to openly discuss their challenges and concerns with the providers [17, 18, 39, 41–44, 48, 50, 52, 57, 58, 66–68, 70, 72, 73, 78, 92–94, 96, 100–102]. Both non-marginalized and socially and culturally marginalized patients, such as indigenous groups, immigrants, and visible minorities stress the importance of their relationship with the provider. Although each population group focuses on different aspects of such relationships, both groups place great value on the patient-physician relationship as a beneficial factor for medication adherence. Marginalized groups describe issues such as language and cultural barriers while non-marginalized groups focus on systemic barriers to building a positive relationship, such as long wait times for short appointments.

Patients consider providers the major and most reliable source of information about their condition and their treatment [19, 38, 39, 43, 48, 49, 52–54, 57–59, 64, 66, 70, 76, 78, 93, 94, 96–98, 102, 104]. However, communication barriers may inhibit collaborative relationships, preventing a shared understanding of treatment and therefore hindering medication adherence [17, 20, 40, 42, 43, 48, 50, 52, 53, 55, 60, 64, 65, 68, 70, 72, 73, 76, 78, 81, 91, 94, 97, 99, 105–108]. Authors describe the barriers to communication between patients and providers as reflecting differences in underlying health beliefs and different desires and understandings of the model of care [17, 20, 40, 42, 43, 48, 50, 52, 53, 55, 56, 59, 60, 64, 65, 68, 70, 76, 81, 91, 93, 96, 98, 99, 101, 105–107]. Patients attribute communication barriers to the way clinicians communicate with them, including providing information that is ambiguous, incomplete, or irrelevant, provider time constraints, and lack of shared decision-making strategies among multiple health care providers [17, 20, 40, 42, 43, 48, 50, 52, 53, 55, 60, 64, 65, 68, 70, 72, 73, 76, 78, 81, 91, 94, 97, 99, 105–108]. Information inconsistency [17, 50, 68, 70, 81, 97, 105] and lack of clear information may result in misunderstandings, and lead patients to use other sources of information [22, 42, 43, 48, 50, 53, 55, 56, 68, 70, 73, 76, 91, 98, 99, 105–108]. Patients find nurses or pharmacists more accessible for information or to answer questions about medication [17, 19, 43, 51, 98, 105]. As briefly mentioned earlier, patients’ language and cultural barriers, as well as their low health literacy levels inhibit communication between patient and provider [20, 42, 43, 50, 55, 56, 59, 64, 70, 72, 78, 93].

Providers, while acknowledging the contributions of a collaborative model of care, address systemic, structural, cultural, and linguistic barriers to patient-provider relationships that impact medication adherence [17, 66, 75, 82–85, 95, 98, 110–112]. In particular, providers recognize different ways in which they may affect patient adherence, including poor “detective work” when devising treatment regimens, poor negotiation abilities, delay in starting insulin therapy, cultural insensitivity, incorrect *a priori* assumptions about patient knowledge and understanding of the treatment, as well as feelings of powerlessness and frustration which affects the healthcare professionals’ ability to provide adequate recommendations [17, 75, 84, 85, 112].

Health care providers also identify patient- related factors affecting their relationship, such as: patients’ passive role, communication barriers, cultural barriers, patients’ distrust in the provider, intentional non-compliance, and patients’ low health literacy levels [20, 83, 84, 95, 110, 112]. Providers describe patients’ passive behaviour as stemming from patients’ negative past clinical encounters, distrust in healthcare providers, deferential attitudes, or patients’ misinformed expectations [20, 83–85, 112]. Most importantly, clinicians value addressing patients’ needs, in order to “figure out” and”fix” reasons for non-compliance [66, 75, 82, 84, 85, 95].

Sometimes providers address the negative impact of structural and language barriers to patient-provider communication – which in turn hinders medication adherence [17, 20, 85, 95, 110]. Systemic problems include long waiting lists, busy schedules, and practice organization barriers, which limit physicians’ available time to communicate with patients [20, 95, 110]. In particular, time constraints and systemic barriers delay their decision to start treatment therapies (e.g. insulin), which need several clinical encounters to adequately instruct patients [84, 95, 110].

### Information & knowledge

Patient accounts of how they negotiate their medication regimens offer explanations for why they choose to manage their condition in a way that suits their personal circumstances and understanding of their health, body, and diabetes [60, 68, 73, 79, 81, 102, 108]. Overall, studies present contradictory findings about the relationship between understanding and adherence [38, 44, 46, 57, 63, 65, 66, 70, 81, 94, 103, 107, 113]. For some patients, a lack of understanding and inadequate knowledge about the medication [47] and prevention of a complication [50, 52, 59] results in poorly controlled blood glucose levels and poor adherence [43, 50, 52, 56, 69, 71, 82, 91, 101]. In other instances, patients report that they understood medications’ importance, but not how the medications work [17, 40, 44, 53, 59, 78, 85, 91, 97, 103]. Thus, patients often report abstaining from medication when asymptomatic, or they consciously decide to take medication according to how they feel [17, 62, 69, 76]. Other studies describe patients as knowledgeable, but unable to translate this knowledge into appropriate action (e.g. “what to do when things go wrong”) [40, 91, 103, 108, 114]. This kind of understanding may improve with experience [44, 51, 94, 97], necessitating a set of problem solving strategies [51, 102, 115], including creative solutions to diabetes self-management [68, 94, 97, 100, 102]. In light of these contradictory findings we may conclude that the role of information and understanding varies in importance depending on individual circumstances.

Patients value the information received by their providers on medication treatment, self-management strategies and on navigating the health care system [19, 38, 43, 48, 52, 57, 70, 78]. Patients also value the information provided by a variety of ancillary resources, such as clinic dieticians and nutritionists and peer support groups [22, 47, 49, 55, 57] and educational programs, including self-management education classes and medication counselling services [19, 38, 48, 55, 65, 68, 70, 92, 97]. Additionally, patients note that they appreciate the opportunity to share information and knowledge, and learn from others who live with the same condition who successfully cope with their condition [22, 45–50, 52, 53, 55, 58, 68, 76, 88, 91]. However, patients also identify the need or desire for more information and management strategies [19, 38, 47–49, 53, 55, 66, 105, 108], especially in language and culture-specific ways [88, 91, 107, 116].

Providers describe patients’ lack of sufficient knowledge about the disease as one of the primary reasons underlying poor medication adherence [17, 84, 110]. As this provider reports, ‘Oh, I probably said that it [the cholesterol] was alright and then she thought is was alright to stop, something like that, that's possible? That happens: they think everything is in order again’ [95].

Providers also report a strong empathy for patients around ideas such as the complexity and impact of diabetes as an unpredictable, frustrating, and long-term disease, identifying the importance of involving and integrating all aspects of the patients’ life [75, 83]. Providers identify the following information priorities for patients: integrated knowledge acquisition about the nature of the disease, medications used and how they work, lifestyle factors (diet, nutrition, exercise), self-care, monitoring procedures, underlying processes of diabetes, and the relationship between diabetes symptoms, medication, and long term consequences [19, 20, 75, 82]. Providers consider discussions about medication-related issues, and improvement of patients’ medication knowledge, important to promoting medication adherence [66, 75, 82, 84, 85, 95]. Providers also recognize language barriers, which limit a more in-depth conversation about a patient's circumstances and health beliefs [20, 110].

### Medication administration

For many patients, the administration of medication poses a significant barrier to adherence. Patients describe fear as a common barrier to insulin administration, in particular fear of needles, fear of consequences of administering insulin incorrectly, and the pain of injection or blood testing [37, 38, 41, 44, 46, 49, 50, 58, 69, 71, 73, 83, 88, 113, 117]. Other patients specifically mention they were not afraid of needles and did not find insulin injection painful [58]. Patients relate insulin administration to other psychological barriers, such as a feeling of stigma around the possession and use of an injectable medication because of the possibility of being mistaken for an illicit drug [37, 38, 41, 50, 58, 68, 73, 88, 100, 102].

According to several studies, co-morbid conditions represent a general barrier to medication administration [17, 22, 50, 57, 58, 66, 68, 77–80]. Patients who take multiple medications may experience forgetfulness, confusion about the purpose, name, and the potential for interactions with other medications [43, 44, 50, 59, 62, 64, 78–80, 99, 105, 117]. The burden of the medication regimen is typically linked to the rigidity of medication which impedes flexibility in every day life, as this patient reports, ‘just the timing and remembering to take your pills on time. It’s a real effort to take them at the right time” [37, 50, 58, 61, 68, 73, 88, 102, 106, 108]. The development of habit-forming routines may encourage medication adherence [59, 108]. When the patient hasn’t established, or has disrupted, the routine, medication adherence declines. This includes minor (e.g. skipping meals) [17, 59, 68, 76, 78, 79, 91, 118], or social and contextual factors in the patient’s life, such as childcare, domestic duties, or work schedules can interfere with patient’s routines [50, 61, 68, 94, 103].

Patients acknowledge family’s instrumental support as a practical means to help integrate the treatment regimen in patients’ daily lives [17, 49–52, 54, 56–59, 66, 68–70, 76, 78, 118]. However, some patients describe fear of being a burden on their family [49, 69, 77] or unsupportive family members as a direct barrier to medication adherence [17, 22, 37, 40, 48, 49, 52, 66, 68, 69, 77, 79, 118].

In general, providers do not recognize the administration of medication as a potential barrier to adherence, except in the case of patients with physical or cognitive impairments [75,82,84], co-morbid conditions [75, 83, 84], or related to treatments, for example fear of needles upon initiation of insulin treatment [20, 82, 84, 86, 110]. Healthcare providers perceive family support as crucial for patients with poor cognitive and physical resources,[82, 110] for reinforcing providers’ medication instructions, and for holding the patient accountable for his/her self-management [17, 82, 84, 110].

### Social and cultural health beliefs

Health beliefs about medication and diabetes are often linked to social or cultural understandings about the body, diabetes and medication, which in turn can affect medication adherence in many different ways. Multiple factors shape these health beliefs, such as the information sources used by the patient, past experiences, attitudes of others, faith and religious beliefs, education, and cultural community [38, 41, 44, 48, 71]. A patient's health belief system may affect the way he or she decides to approach medication adherence, and how to integrate (or not) the requirements of the medication regimen into everyday life [44, 76].

A patient's health beliefs and cultural background will also affect the relationship s/he desires with the prescribing physician [44, 48, 50, 59, 60, 62, 69, 71, 72, 74, 89, 91, 117]. For instance, patients who are members of historically oppressed communities by the dominant culture can be suspicious of medical advice [69, 71, 74, 89, 117]. Patients from cultures that perceive physicians as high status individuals with significant authority may feel uncomfortable asking questions [20, 93, 98]. Several papers recommend including the patient in a culturally appropriate way as an active partner of care to improve medication adherence [39, 41, 57, 58, 70, 93]. However, providers should adapt to the patients’ beliefs and preferences, as some patients may refuse to work with clinicians in this way.

Social and cultural beliefs also affect patient preferences for allopathic (Western biomedicine) compared to traditional medications. Some patients express their intention to take them alongside traditional medications [53, 56, 62, 74, 89]. Many patients indicate a preference for traditional or herbal medications, and a suspicion or distrust of allopathic medication [20, 44, 53, 62, 69, 79, 83, 88, 89, 92, 94, 116, 117]. These patients describe allopathic medicine as unnatural [38, 44, 78, 79, 88, 92], the cause of side effects and complications [20, 53, 62, 78, 88, 92, 117], incongruent with their understanding of holistic health [20, 79, 88, 94]. In contrast, patients describe traditional remedies as effective [88, 89, 92, 116], a link to their past and present cultural communities [71, 88] and easier to access [20, 88, 89].

Provider perspectives rarely address the issue of traditional medication alongside or instead of allopathic medication [95]. Providers are more likely to mention challenges linked with the patient's cultural background and beliefs, such as aversion to insulin, fatalistic attitudes, the perception that fat is healthier or a desire to please the physician [20, 83, 84, 110, 112].

### Financial issues

Patients widely mention the cost of medication as a barrier to medication adherence [17–19, 37, 42, 44, 46, 50, 64, 68, 70, 75, 76, 81, 89, 91–93, 97, 119], although studies involving participants with access to public health insurance less likely to mention cost as a barrier [47, 73]. Financial barriers can extend beyond the cost of medication and physician services. Even patients with health insurance can struggle to afford testing supplies, syringes, and non-physician supportive care [17, 19, 37, 70, 75, 76, 81, 91, 92, 97]. Patients living in poverty also face other structural and material constraints such as low health literacy, poor quality housing, shift work, stress, inability to access healthy food etc., that affect their ability to adhere to medication regimens [37, 68, 70, 89, 119]. When faced with financial constraints, patients may use tactics including: taking medication less often than recommended, choosing the most “important” medication to pay for, sharing pills with other people, drawing on personal capabilities and social networks, and asking their doctor for help [18, 50, 68, 89, 92].

Providers interviewed in some projects understand the financial barriers that patients may face, but commonly do not identify this issue as a barrier to medication adherence [17, 18, 75, 83, 112]. In some cases, while providers may recognize that some patients struggle with the cost of medication, ‘these were clearly secondary concerns from the clinicians' perspective’ [18]. In several instances, clinicians acknowledge the cost of medication as a contextual barrier along with other struggles related to low socio-economic status [83, 112]. While some clinicians perceive that these struggles are outside of their realm of influence [75], others offer creative strategies for alleviating financial burden such as prescribing generics, giving samples, changing the regimen to accommodate constraints or helping patients participate in patient assistance programs [18, 75].

## Discussion

Extensive qualitative research exists on the topic of barriers to medication adherence amongst community-dwelling adults with Type 2 diabetes. Our synthesis of this research to date suggests that providers and patients share some common understandings of these barriers, as well as facilitators to overcome them. However, the qualitative research also identifies many points of misunderstanding, miscommunication, and missed opportunities for intervention. In general, providers tend to limit their focus to clinically-oriented issues, while patients describe a much wider range of problems with medication adherence that arise from the personal, social, and practical challenges of living with diabetes. To the extent providers understand and address these wider concerns (possibly through a multidisciplinary professional, patient-centered approach to care), they will potentially improve both medication adherence and patient experiences. This reflects what the literature addresses as patient-models of care, where patients *adhere* to medications and not *comply* (emphasis added) overtaking past paternalistic models of care [6, 16, 120].

Our synthesis on medication adherence may contribute to define more clearly the key dimensions of the person-centered (PC) model of care and illustrate how this model may improve medication adherence among Type 2 diabetic patients. According to Bower and Mead [16], PC care is based on the following dimensions: inclusion of biopsychosocial factors, viewing the patient as a person, enhancing patient’s empowerment and autonomy, involving the patient in the decision-making process through a two-way communication process and negotiation, encouraging a collaborative and mutual trusting relationship between patient and provider, and emphasizing the doctor as a person.

Our study shows that patients and providers often agree on the importance of medication adherence for symptom improvement, the benefit of a collaborative and responsive relationship between patient and provider, and effective communication of information. The integration of patient’s perspectives in the clinical relationship, based on a mutual and trusting relationship, broadens the scope of the explanatory model of illness by addressing different ‘dysfunctional’ states and the possible interventions areas to improve adherence [7, 10, 16, 120–122]. As the PC model of care describes, these factors help patients integrate their medication regimen into their own system of health beliefs, the individual context of their everyday lives, and their changing circumstances [10, 16, 120, 121, 123, 124].

Conversely, patients commonly cite providers’ lack of collaboration, lack of interest in the patient’s life and context, poor communication, or time constraints as interfering with their medication adherence. Providers on the other hand, as documented in recent studies [125–127], tend to address systemic barriers, such as limited time consultations and lack of inter-professional collaboration, as obstacles challenging the prioritization of patients’ medical and psychosocial needs.

Providers and patients differ significantly on how best to influence patients’ self-management practices. Providers tend to focus on patients’ knowledge about the physiology of the disease and role of medical and lifestyle interventions: i.e., the nature of the problem, what needs to be done, and how. While some providers do recognize the importance of emotions and psychosocial factors, providers relate these more to the motivation, than to the capacity, to use medication properly. Recent studies corroborate these findings, indicating that providers consider motivation as crucial for patients’ understanding of the illness and effective medical education [126, 128].

Patients, however, describe diabetes and medication self-management as a multi-dimensional experience. Practical aspects include finances, daily routines, and the need for instrumental support. Psychosocial aspects include health beliefs, emotional impacts, social and cultural understandings of diabetes and medications. Self-management models based on behaviour and integration theories reflect this perspective on medication adherence behaviours and self-care activities, acknowledging both the psychosocial and biomedical nature of the medication regimen [129, 130] and highlighting that perceptions may differ between patients and providers on treatments goals and strategies [131]. Providers’ focus on biomedical problem-solving can also leave patients feeling “reduced to their disease”. This runs counter to the person- or patient-centered care approach that calls for treating people holistically, with their attention to their disease placed in context of attention to other factors in their lives [16, 121, 123]. All of these factors influence the way that patients interpret and apply medical advice.

This synthesis of the qualitative research on patient and provider perspectives underscores the recommended shift from the traditional medical view of medication “compliance” to the more patient-centered view of medication “concordance” with patients’ many other needs, pressures and demands [7, 10, 13, 16, 121, 123].

### Strengths and the limitations

A number of strengths and limitations of this study are worth noting. First, this study provides an updated and comprehensive qualitative systematic synthesis of Type 2 diabetes patients’ and providers’ different perspectives on medication adherence. Existing systematic reviews of qualitative research focus on diabetes management more broadly [122, 132–134], and quantitative reviews on medication adherence elicit different types of information. For example, quantitative studies concentrate on measuring medication adherence rates among Type 2 diabetes patients, or measuring medication adherence rates for specific drug therapies, nutrition regimens and educational interventions designed to improve adherence, rather than addressing the reasons and experiences of trying to adhere to medication regimes [12, 135–139]. By comparing patient and provider perspectives on this issue, we are able to provide an interpretive synthesis of the constellation of challenges that patients may face when prescribed a medication regime for their diabetes.

Another strength of this study is the large body of qualitative research available for synthesis on this topic: we were able to include 86 studies that together captured thousands of patients’ experiences. The integrative meta-synthesis method also allowed us to distil robust thematic findings, each supported by a number of studies and therefore more transferable across settings. The rigor of the synthesis allows establishing generalizability and consistency of the findings among a large number of studies and across different countries, patient population groups, and patient demographic differences. A significant portion of the included articles (33 out of 86) focused on very specific populations of diabetic patients, such as indigenous groups, immigrants, and minorities. We used a constant comparative technique to examine medication adherence barriers across such groups in our analysis and found our main categories to be consistent across groups, with some variation in the sub-categories. For example, we noted the consistency of the theme of the importance of the patient-provider relationship across studies that included marginalized and non-marginalized populations. Comparative analysis of this theme revealed that the patient-physician relationship was consistently mentioned as important by authors of both types of studies, although they tended to focus on different aspects of this relationship. For example, marginalized participants spoke about the difficulty in communicating with language and cultural barriers and the impediments that this provided to a strong, supportive relationship with their physician. Non-marginalized participants were more likely to focus on barriers such as wait times and short length of appointments. Both groups emphasized the importance of this relationship on their ability to self-manage their diabetes. Similarly, the results did not present consistent differences by age or gender across study populations, as the populations were overall consistent in terms of age and gender. We found that barriers to medication adherence were consistent across the different diabetic and demographic populations considered in the 86 studies. Therefore we are not presenting the results as stratified along these lines. Findings concerning culture in particular may be less transferable to jurisdictions and cultures not captured in this body of research. A limitation of this study is its focus on English-language research reports.

This study was limited also in other ways. First, this review includes only research conducted between 2002–2013. These dates reflect an attempt to include a manageable body of current literature. Given the depth and complexity of qualitative data, 86 studies provide a large body of data to describe and interpret. The WHO report on medication adherence published in 2003 [1] offers an authoritative summary of the state of knowledge before 2002; we have provided a review of this foundational literature in the introduction of the current manuscript.

Second, this meta-synthesis retrieved a great number of patient experiences reflecting a limitation of the underlying body of research: the relatively fewer qualitative research into providers’ perspective on patient-barriers to medication adherence among Type 2 diabetes patients. Recent studies corroborate our results reinforcing the sense of saturation of our data [125–128, 131], however, because studies on patient, not provider, perspectives continue to dominate the field, we highlight providers as an important population for future qualitative investigation and possibly multi-methodology research syntheses.

## Conclusion

This study synthesized Type 2 diabetes patients’ and providers’ views about medication adherence, highlighting that these groups have different medication adherence priorities. While providers tend to focus on patients’ motivations and medication administration practices, patient accounts emphasize their experience of diabetes as part of a holistic consideration of their whole lives. Providers shared clinically oriented perspectives, detailing rich and sophisticated conditional reasoning about their efforts to persuade patients to adhere to their medication regimen. Patients’ accounts of medication adherence describe individual experiences of diabetes medication that are deeply embedded within the context of the individual’s particular life circumstances, emphasizing that medication self-management practices are built upon more than just knowledge and motivation for change. The conceptual divide between patients and providers on the topic medication adherence enriches our understanding of why medication adherence may be experienced as an intractable issue by both patients and providers. The findings of this synthesis may assist providers in identifying potential factors that affect a particular patient’s medication practices. Taking a patient-centered approach to medication self-management may encourage increased understanding the priorities and experiences patients, encouraging providers to identify the multiple underlying factors that promote or inhibit medication adherence in their patients creating the opportunity for patients to voice their questions or concerns about their medication regimens. Interventions that aim to improve medication adherence will benefit from considering the issue of adherence from a patient-centered model of care by tailoring the medication regimen to patients’ life contexts, preferences and self-management practices.
